# Supervised pharmacy student-led medication review in primary care for patients with type 2 diabetes: a randomised controlled pilot study

**DOI:** 10.1136/bmjopen-2015-009246

**Published:** 2015-11-04

**Authors:** R P Adams, G Barton, D Bhattacharya, P F Grassby, R Holland, A Howe, N Norris, L Shepstone, D J Wright

**Affiliations:** 1 School of Pharmacy, University of East Anglia, Norwich, Research Park, Norwich, UK; 2 Norwich Medical School and Norwich Clinical Trials Unit, University of East Anglia, Norwich Research Park, Norwich, UK; 3 School of Pharmacy, University of Lincoln, Lincoln, UK; 4 Norwich Medical School, University of East Anglia, Norwich Research Park, Norwich, UK; 5 School of Education & Lifelong Learning, University of East Anglia, Norwich Research Park, Norwich, UK

**Keywords:** EDUCATION & TRAINING (see Medical Education & Training)

## Abstract

**Objective:**

To pilot and feasibility-test supervised final year undergraduate pharmacy student-led medication reviews for patients with diabetes to enable definitive trial design.

**Method:**

Third year pharmacy students were recruited from one UK School of Pharmacy and trained to review patient's medical records and provide face-to-face consultations under supervision while situated within the patient's medical practice. Patients with type 2 diabetes were recruited by postal invitation letter from their medical practice and randomised via automated system to intervention or usual care. Diabetes-related clinical data, quality of life, patient reported beliefs, adherence and satisfaction with medicines information were collected with validated tools at baseline and 6 months postintervention. The process for collecting resource utilisation data was tested. Stakeholder meetings were held before and after intervention to develop study design and learn from its implementation. Recruitment and attrition rates were determined plus the quality of the outcome data. Power calculations for a definitive trial were performed on the different outcome measures to identify the most appropriate primary outcome measure.

**Results:**

792 patients were identified as eligible from five medical practices. 133 (16.8%) were recruited and randomised to control (n=66) or usual care (n=67). 32 students provided the complete intervention to 58 patients. Initial data analysis showed potential for impact in the right direction for some outcomes measured including glycated haemoglobin, quality of life and patient satisfaction with information about medicines. The intervention was found to be feasible and acceptable to patients. The pilot and feasibility study enabled the design of a future full randomised controlled trial.

**Conclusions:**

Student and patient recruitment are possible. The intervention was well received and demonstrated some potential benefits. While the intervention was relatively inexpensive and provided an experiential learning opportunity for pharmacy students, its cost-effectiveness remains to be determined.

**Trial registration number:**

ISRCTN26445805; Results.

**Strengths and limitations of this study**

- The study followed recommendations published by the Medical Research Council for implementation of feasibility and pilot studies.
- The intervention was developed with significant stakeholder involvement, a range of primary outcome measures were tested for suitability and the process for collecting resource utilisation data was identified.
- Self-selection bias was found within those students who consented to participate.
- The trial was unblinded with the service providers, patients and research all aware of group allocation and intervention content.
- The large number of statistical tests carried out creates the possibility that findings could be false positives and as this was a pilot and feasibility study which, therefore, used a low power we could potentially fail to detect small to moderate sized effects.

## Introduction

It is estimated that preventable harm from medicines costs the National Health Service (NHS) in England £750 million per year.^1^ A systematic review in 2009, based on UK research, estimated that only between 4% and 21% of patients achieved the optimum benefit from their medication.^2^ A systematic review of publications between 1966 and 1999 reported the prevalence of preventable drug-related admissions to hospital as 4.3%.^3^ Additionally, patients not taking their medicines as agreed with the prescriber has been reported to cost the UK NHS an estimated half a billion pounds a year.^4^ Consequently, interventions designed to reduce adverse drug events and improve patient medicines-taking behaviours are required.

Medication review (MR) has been defined as ‘a structured, critical examination of a patient’s medicines with the objective of reaching an agreement with the patient about treatment, optimising the impact of medicines, minimising the number of medication-related problems in addition to reducing waste.^5^ MR in the UK was described as operating at four different levels,^5^ with the key component of level 3 medication reviews being the involvement of the patient while accessing medical records.

Pharmacist-led medication reviews have been shown to reduce costs associated with unnecessary prescribing of medication while potentially providing patient benefit.^6^ However, recent UK-based research utilising pharmacists to provide medication reviews found a counter-intuitive increase in hospitalisation,^7^ with one partial explanation being the didactic nature of the pharmacist communication.^8^ ^9^ Effective communication skills are necessary to improve patient behaviours in terms of lifestyle^10^ and medication taking.^11^ Consequently, models of consultation behaviour have been developed and are commonly used within the education of healthcare professionals.^12^ ^13^

While UK pharmacy graduates develop expertise in the pharmacology and therapeutics of medicines, undergraduate training currently lacks significant patient contact. This provides limited opportunities for the development of clinical and communication skills during training.^14^ In contrast, medical students routinely work with patients during their undergraduate training^15^ and undergraduate students within Schools of Dentistry and Optometry provide services to patients under the supervision of clinical tutors to improve both their clinical and communication skills.^16^ ^17^

Involvement of pharmacy students in the provision of healthcare services in other countries has been reported^18^ ^19^ with very good prescriber acceptance of student recommendations.^20^ However, pharmacy student provision of medication review to patients in the UK, with the dual aims of demonstrating patient benefit and improving student communication and clinical skills, has not been tested. For new models of care to be adopted, it is necessary to demonstrate that they are likely to be cost-effective. There is currently no evidence of this for the provision of pharmacy student-led medication review services.

Two and a half million of the UK population currently have type 2 diabetes with an estimated direct cost to the health system of £8.8 billion/annum.^21^ Pharmacist-led medication reviews for patients with diabetes have demonstrated significant reductions in blood pressure^22^ ^23^ and glycated haemoglobin (HbA1c),^24^ ^25^ both of which are necessary to reduce long-term morbidity.^26^ Approximately 60% of patients are achieving the target HbA1c below 7.5% (59 mmol/mol). 27 Owing to the availability of clearly defined national treatment guidelines^28–30^ and increasing numbers of patients with type 2 diabetes, medication review for patients with diabetes is a health service which pharmacy students may be able to usefully provide.

A pilot and feasibility study was, therefore, undertaken in line with national guidance for the evaluation of complex interventions^31^ to determine recruitment and attrition rates for students and patients; describe the suitability of using student volunteers; test data collection for a future cost-effectiveness analysis; describe the potential effects of the intervention; identify the most suitable primary outcome measure; and estimate variance around this to enable a future trial to be effectively powered.

## Method

ISRCTN No 26445805 was obtained retrospectively rather than prospectively due to misinterpretation of an National Institute for Health Research (NIHR) email stating that “NIHR Clinical Research Network (CRN) Coordinating centre had developed a process which enables automatic registration of all new NIHR Clinical Research Network Portfolio studies via the Portfolio.” We did not notice that they provided details of ISRCTN registration via UKCRN Portfolio, later in the same email.

### Student recruitment and preparation

All 84 third year pharmacy students from one UK school of pharmacy were invited to participate in June 2011. The intervention was timetabled to occur during the final year (fourth) of their undergraduate studies. Informed consent was obtained from students agreeing to participate. A reserve list was created and used to resolve any anticipated drop-out caused by the additional workload created by the study within the students’ final year.

Standard education process already included basic training in consultation skills, data governance and observation of a primary care doctor in practice. In addition, participating ‘study students’ undertook four half-day training sessions comprising the following:

- Use of medical practice information systems using ‘dummy’ patient records;
- Revision of ‘pharmaceutical care planning’ using a care plan designed to enable recording of patient details and also to provide a guide to their consultations;
- A consultation skills workshop which included utilising dummy primary care patient records and training in basic behaviour change counselling;
- Two medicines-related consultations with pre-prepared and scripted professional role-play actor ‘patients’, following which individual and group feedback was provided.

All student activities connected with this trial were undertaken outside the university curriculum, with students donating their time. Student preparative training was undertaken at the University of East Anglia, apart from training in the use of computerised medical records which was provided by NHS Norfolk and Waveney.

Examination results for students (both participating and non-participating) were obtained at the end of year three (ie, shortly after recruitment) to identify potential student self-selection bias.

### Medical practice and patient recruitment

Five Norfolk-based medical practices were purposively recruited by NHS Norfolk and Waveney. The inclusion criteria were:

- Pharmacist working as a prescribing advisor within the practice;
- Using the SystmOne IT medical record system;
- Over 200 patients registered with type 2 diabetes.

Presence of a pharmacist was required to enable student supervision; the same system facilitate student training. A target number of patients was required to increase the opportunity for meeting recruitment targets.

### Patient recruitment

Patients in each medical practice who met the following criteria were posted a letter from the practice asking them to participate in the study:

### Inclusion criteria

- Prescribed non-insulin medication for type 2 diabetes mellitus for at least 2 years to increase likelihood that therapy is stabilised.

### Exclusion criteria

- Deemed unsuitable for inclusion in the trial by their medical practitioner for any reason;
- Enrolled into other clinical trials;
- Diagnosed with a terminal illness.

All recruited patients were randomised to intervention or control (standard care) using an automated randomisation system, developed and controlled by the clinical trials unit, which ensured concealed allocation. Randomisation was undertaken in blocks of four to maximise equality of group size. All researchers and clinical staff involved with generating outcome date were blind to participant allocation.

As a pilot study it was decided to test a number of potential primary outcome measures, with results enabling a decision as to which measure to use in a future study:

- HbA1c;
- Blood pressure;
- Lipid profile.

Secondary outcome measures were the effect on:

- Health-related quality of life (EQ-5D);
- Satisfaction with information about medicines (SIMS);^32^
- Medication adherence (MARS);^33^
- Patient's beliefs about medicines (The Beliefs and Medicines Questionnaire);^34^
- Diabetes Treatment Satisfaction Questionnaire (DTSQ).^35^

Level 2 medication reviews were undertaken by students between November 2011 and February 2012 while student-led patient consultations (level 3 medication review) took place between December 2011 and March 2012.

### Sample size justification

As a pilot study it was not appropriate to power the study. Consequently we determined the likely precision of the study for estimating the effect of the intervention on the continuous end points by estimating the expected value of the half-width of the 95% CI around the difference in means between the intervention and control groups. Assuming 80 patients in each group and a SD of 1.5% in the primary outcome variable of HbA1c then the estimate from this pilot study of the effect of the intervention on this end point would be within 0.5% of its ‘true’ value. An observed difference in group means >0.5% (eg, HbA1c 7% compared to 7.5%) would be statistically significant.

From each of the five practices, 32 patients were, therefore, required providing 160 in total (80 in each of intervention and control arms). It was planned that each student would be allocated two patients in the intervention arm for review and consequently 40 students were required.

### Baseline data

Both intervention and control participants were posted a questionnaire comprising the secondary outcome measures, which included an additional question asking whether they used a medicines compliance aid.

The patient notes within the medical practice were investigated to obtain:

- Demographic data;
- Most recent results prior to recruitment for HbA1c, blood pressure and lipid profile;
- Medication utilisation (cost of all prescriptions issued for each patient for a baseline period of 3 months prior to intervention and 6 months postintervention;
- NHS resource utilisation calculated as cost (using standard NHS staff cost) of all NHS contact for a baseline period of 3 months prior to intervention and 6 months post-intervention in primary and secondary care.

The last two items were included to determine the feasibility of collecting data for a future cost-effectiveness analysis within a randomised controlled trial.

### Intervention

At the patient's medical practice, students were randomised to work in pairs. This provided additional support and shared learning in addition to the wider clinical experience provided by access to additional patient records. Each pair, therefore, worked to undertake level 2 medication reviews for four intervention patients under the supervision of a PCT pharmacist. Students compared prescribing with national guidance^28–30^ and created individualised pharmaceutical care plans (PCP) on a predefined pro forma.

PCPs included demographics, allergies, special needs, lifestyle for example, diet and smoking status, relevant medical history, medication adherence, pharmacy use, current prescribed medication, over the counter, herbal and homeopathic preparation use and any care issues identified. After discussion and agreement with the supervising pharmacist, care issues identified were fed back on a custom-designed form, to either the patient's specialist diabetes nurse or medical practitioner who then decided on the final action.

Patients were then offered a range of flexible dates and times, to maximise participation of a meeting between each student and an individual patient.

The presence of a supervising pharmacist was ensured during all student-led medication reviews to ensure competence. The consultation (level 3 medication review) took place at the patient's medical practice and was separated by at least 2 weeks from the level 2 review to enable the doctor or nurse to rectify or question any problems identified by students, prior to the subsequent student-led consultation. As planned in the protocol, each student provided two patient consultations which had no time limit imposed on their duration.

### Follow-up

Six months postintervention, a questionnaire was posted to both intervention and control participants. The questionnaire was identical to the baseline version but with an additional question regarding frequency of community pharmacy service utilisation. Intervention participants were also asked to report any change in utilisation of community pharmacists following the student consultation to identify any change in attitude to or view of community pharmacists. The same data as baseline were collected from the medical practice, with HbA1c, blood pressure and lipid profile collected at 6 months postintervention with a cut-off point of 1 year; and medication and resource utilisation for the 6-month period postintervention.

### Stakeholder meetings

Stakeholder meetings were held with patients, students, primary care pharmacists and medical practice staff both before and after the intervention. Interested individuals were recruited prior to the study's implementation to inform the design of the intervention and trial, while study participants were recruited postintervention to learn from their experiences. All meetings were recorded and transcribed verbatim.

### Data analysis

The variance in the difference between each primary or coprimary outcome measure between groups was undertaken on an intention-to-treat basis using independent t test and Mann-Whitney U test. The patient recruitment rate and the medicine-related consultation uptake rate were both determined. SPSS V.22 was used for analysis.

### Stakeholder meeting analysis

Simple content analysis which proceeded to identify all themes, as recognised by the researcher, was undertaken for development meetings with the main learning points recorded for action as appropriate. Review meeting analysis followed the general principles of the framework approach.^36^ There was no independent analysis of the data. Nvivo 10 was used to facilitate analysis.

### Health economics analysis

As a pilot study we report completion rates for resource use items and the EQ-5D. Additionally, unit costs^37^ were extracted in order to enable the cost of the intervention to be estimated.

## Results

All five medical practices which responded to recruitment requests were consented to join the study.

**Figure 1:**
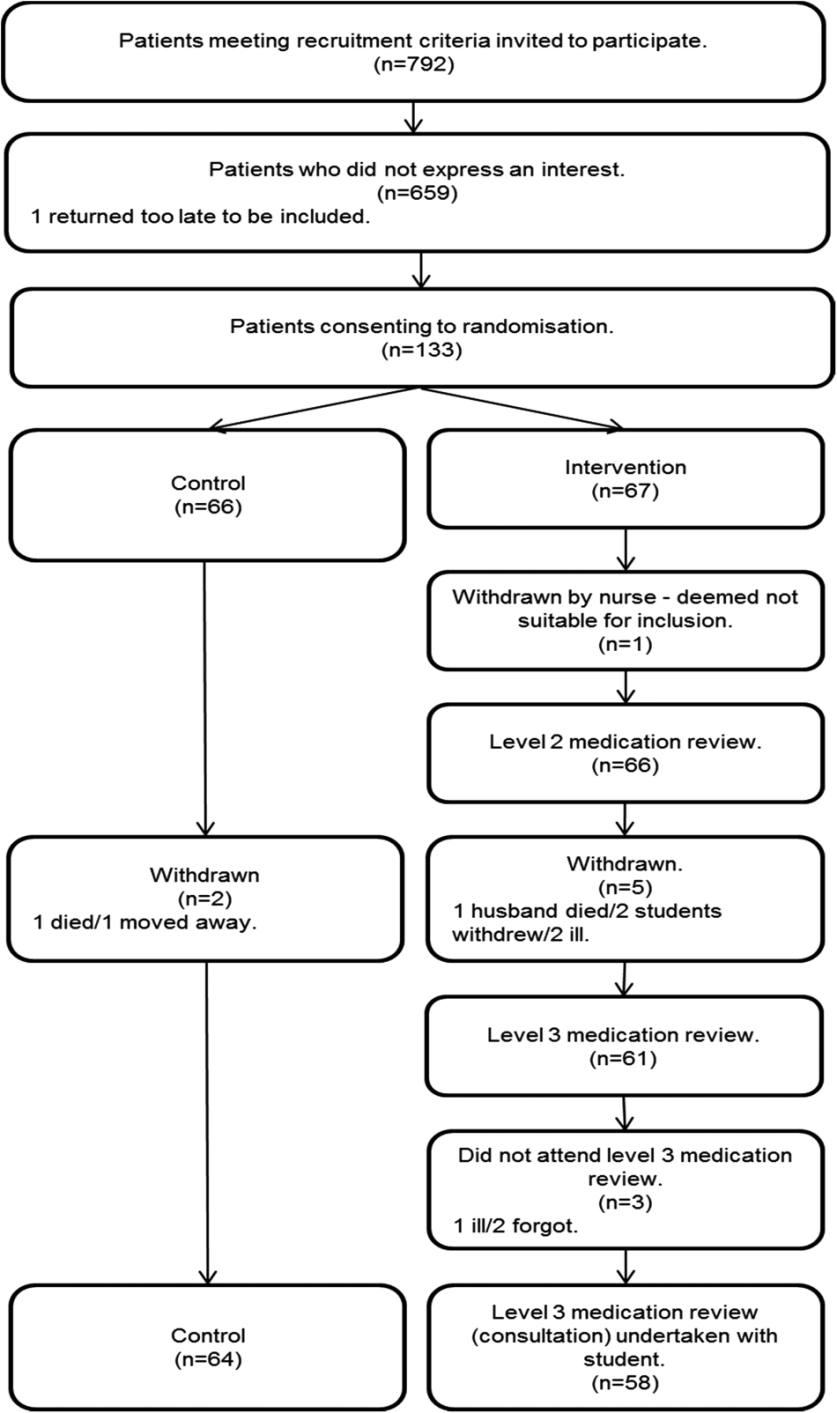
Consort diagram for patient recruitment.

Figure 1 summarises the patient recruitment process which achieved a 16.8% consent rate. Recruitment rates within the five medical practices were 17.2%, 19.6%, 20.1%, 18.5% and 9.4%. The research team received a number of contacts from patients who had been approached by the medical practice with a 9.4% response rate to complain that they had received recruitment letters without stamps. The first two practices recruited in time to enable students to complete medication reviews before Christmas 2011. The other three practices recruited later resulting in student-led medication reviews in one practice being undertaken in March 2012. Of the 67 patients randomised to intervention, 91% remained in the study prior to the student-led medication review with 100% of those agreeing to an appointment with a student for a consultation. Of these, three (4.9%) failed to attend the consultation.

**Figure 2:**
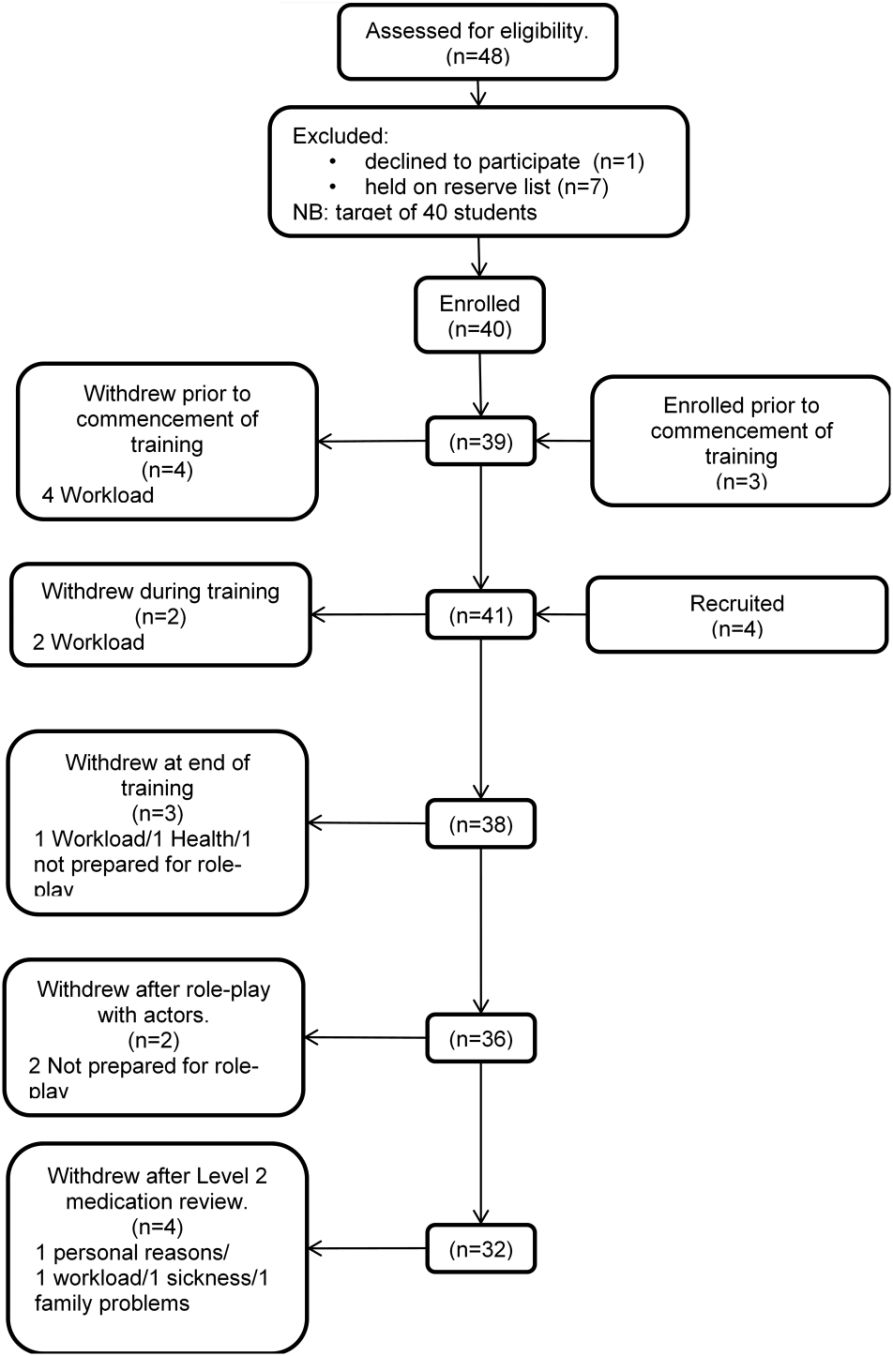
Consort diagram for student participants.

Figure 2 provides a summary of the flow of students at each stage of the study. Of the 47 students who volunteered and completed a consent form, 12.75% were male. The first 40 students volunteering were recruited with the remaining seven forming the reserve list used if recruited students left the study. Patient consultations were undertaken by 32 of the 47 (68%); however, of these six only undertook one consultation due to patient numbers. Mean (SD) examination scores at end of year three (at recruitment) for participating and non-participating students were 62.80 (7.91) and 58.91 (7.98), respectively. Academic performance of participating students was significantly better than non-participating students at the point of recruitment (p<0.05, independent samples t test).

Table 1 presents baseline, demographic, clinical and questionnaire data for control and intervention patients. The data indicate that randomisation resulted in reasonably comparable groups, although the wide SD demonstrates considerable numbers of patients in each group were failing to achieve clinical targets.

**Table 1:** Comparison of patient demographics at baseline

Characteristic	Measure	Usual range or ideal figure	Intervention patients (n=67)	Control patients (n=66)
Age	Mean (SD)	NA	69.18 (10.46)	68.31 (9.46)
Male	Number (%)t	NA	45 (68%)	38 (58.5%)
HbA1c mmol/mol	Mean (SD)	48	56.81 (11.12)	59.71 (13.92)
Total cholesterol mmol/L	Mean (SD)	<4.0	4.14 (0.99)	4.19 (0.91)
Blood pressure mm Hg				
Systolic	Mean (SD)	140	132.48 (11.98)	131.65 (10.90)
Diastolic	Mean (SD)	80	73.22 (8.15)	72.13 (9.54)
Euroqol VAS scale	Median (IQ)	NA	(n=45) 80 (70, 90)	(n=48) 80 (70, 90)
EQ-5D-5L	Mean (SD)	NA	(n=43) 0.766 (0.168)	(n=48) 0.736 (0.184)
SIMS			(n=43)	(n=47)
Total	Median (IQ)	17	12 (7, 17)	12 (8, 15.5)
Action and use	Median (IQ)	9	7 (4.75, 9)	7 (5, 9)
Potential problems	Median (IQ)	8	5.5 (2.25, 8)	5 (2, 8)
BMQ			(n=43)	(n=47)
Necessity	Median (IQ)	5	18 (16, 21)	19 (17, 21)
Concerns	Median (IQ)	5	11.5 (10, 14)	13 (10, 16)
MARS			(n=43)	(n=47)
	Median (IQ)	25	24 (23, 24)	24 (23, 24)
DTSQ			(n=45)	(n=48)
Treatment satisfaction	Median (IQ)	36	30 (26, 35)	31 (26, 34)
Problem-hyperglycaemia	Median (IQ)	0	1 (0, 3)	2 (0, 3)
Problem-hypoglycaemia	Median (IQ)s	0	0 (0, 1)	0 (0, 3)
Using a medicine compliance aid (MCA)	Number (%)	NA	44 (47.7%)	48 (43.8%)

DTSQ, Diabetes Treatment Satisfaction Questionnaire; Euroqol VAS scale, Euroqol visual analogue scale; HbA1c, glycated haemoglobin; MARS, medication adherence; SIMS, satisfaction with information about medicines; NA, not applicable.

Table 2 presents 6 months postintervention data including clinical data and results of patient-completed questionnaires. Questionnaire responses were received from 94 (70.7%) of the 133 patients with non-respondents and omission of responses to individual questions being comparable across the two groups. Significant differences between intervention and control groups were only observed for change in quality of life and some elements of SIMS.

**Table 2:** Comparison of patient outcomes post-intervention

Characteristic	Measure	Intervention (n=67)	Control (n=66)	p Value	Mean (95% CI) difference OR Median difference
HbA1c mmol/mol	Number (%)	59 (88.1)	59 (89.4)		
	Mean (SD)	56.32 (11.5)	59.68 (13.2)	0.14#	−3.36 (−7.781 to 1.11)
Total cholesterol mmol/L	Number (%)	61 (91.0)	53 (80.3)		
	Mean (SD)	4.22 (1.0)	4.01 (0.8)	0.47#	0.13 (−0.23 to 0.5)
Blood pressure mm Hg	Number (%)	61 (91)	60 (90.9)		
Systolic	Mean (SD)	132.26 (12.9)	127.98 (11.9)	0.06#	4.35 (−0.15 to 8.84)
Diastolic	Mean (SD)	73.38 (6.8)	70.97 (9.5)	0.11#	2.41 (−0.52 to 5.34)
Euroqol VAS	Number (%)	51 (76.1)	48 (72.7)		
	Median (IQ)	80 (70, 85)	72.5 (61.3, 85)	0.182*	7.75
Change from baseline	Number (%)	37 (55.2)	40 (60.6)		
	Mean (SD)	+2.00 (8.73)	−6.24 (18.28)	0.015#	8.24 (1.65 to 14.8)
EQ-5D-3L	Number (%)	51 (76)	46 (69.7)		
	Mean (SD)	0.768 (0.224)	0.736 (0.233)	0.49	0.031 (−0.06 to 0.123)
Change from baseline	Number (%)	35 (52.2)	38 (57.6)		
	Mean (SD)	0.048 (0.133)	−0.003 (0.134)	0.103#	0.052 (−0.011 to 0.114)
SIMS	Number (%)	50 (74.6)	47 (71.2)		
Total	Median (IQ)	14 (9.2, 17)	10 (6, 15)	0.073*	4
Action and usage	Median(IQ)	8 (7, 9)	8 (6, 9)	0.078*	0
Potential problems	Median (IQ)	3 (5, 8)	2 (3.5, 7.7)	0.037*	1
BMQ	Number (%)	48 (71.6)	49 (74.2)		
Necessity	Median (IQ)	20 (18, 22.5)	20 (19, 22)	0.925*	0
Concerns	Median (IQ)	14 (12, 16)	14 (12, 15)	0.825*	0
MARS	Number (%)	50 (74.6)	48 (72.7)		
	Median (IQ)	24 (23, 2)	24 (23, 2)	0.843*	0
DTSQ	Number (%)	49 (73.1)	48 (72.7)		
Treatment satisfaction	Median (IQ)	32 (26.5, 35)	30.5 (27.7, 33.2)	0.413*	1.5
Problem-hyperglycaemia	Median (IQ)	1 (0, 3)	1 (0, 2)	0.360*	0
Problem-hypoglycaemia	Median (IQ)	1 (0, 1)	0 (0, 2)	0.929*	1
Using a medicine compliance aid (MCA)	Number (%)	50 (74.6)	46 (69.7)		
	Number (%)	23 (46.0)	25 (54.3)	0.540$	

The test used to identify the p value is indicated by # Independent samples t test, * Mann Whitney U, $ Fisher's exact test.

BMQ, Beliefs about medicines questionnaire; DTSQ, Diabetes Treatment Satisfaction Questionnaire; Euroqol VAS scale, Euroqol visual analogue scale; HbA1c, glycated haemoglobin; MARS, medication adherence; SIMS, satisfaction with information about medicines.

**Figure 3:**
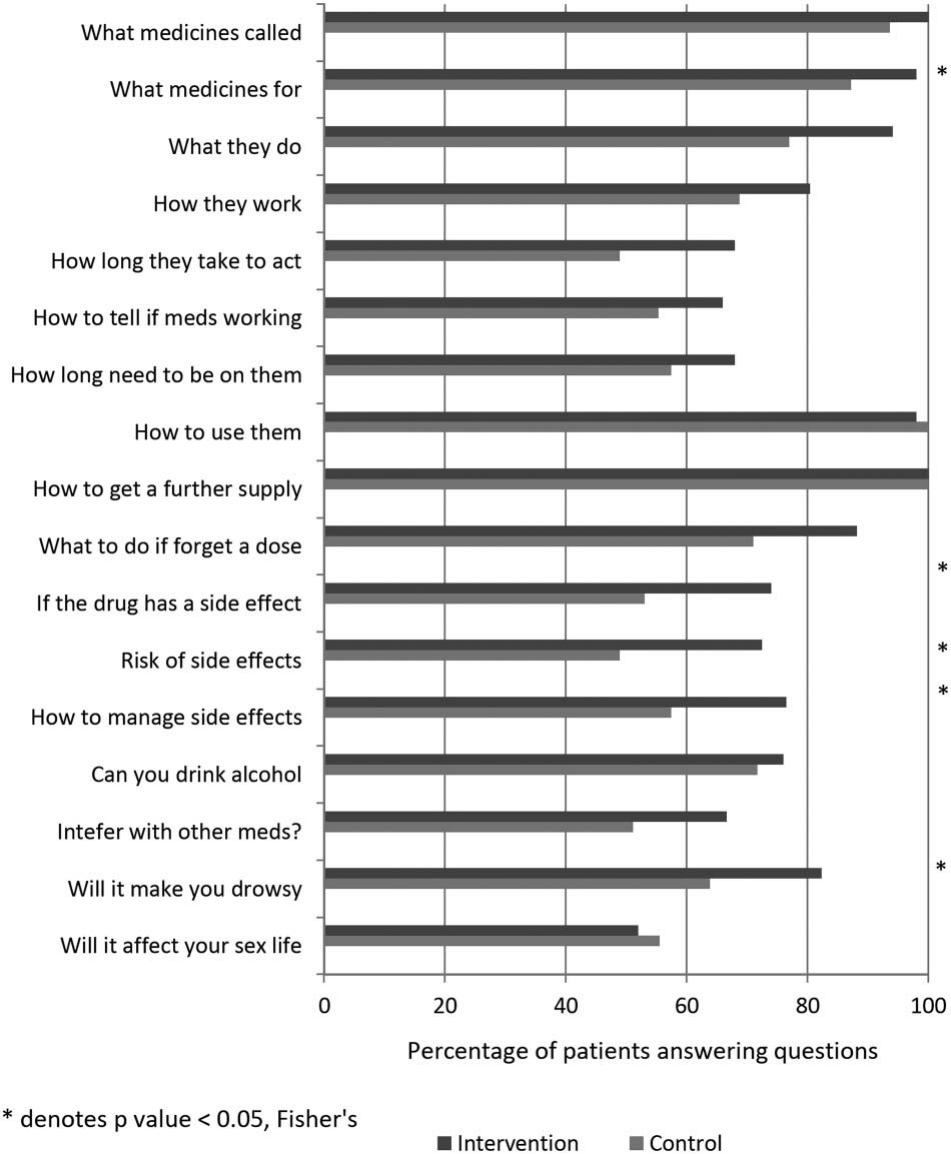
Provides a comparison of the satisfaction with information about medicines (SIMS) questionnaire responses for intervention and control patients at follow-up (6-month postintervention). It demonstrates that patients in the intervention group were significantly more satisfied with five parameters (one action and usage, four concerns) of the 17 questions (control n= 34; intervention n= 36).

Figure 3 illustrates a comparison of the SIMS questionnaire responses for intervention and control patients at follow-up (6 months postintervention). It demonstrates that patients in the intervention group were significantly more satisfied with five parameters (one action and usage, four concerns) out of the 17 questions.

Control n=34 Intervention n=36.

Twenty-five (55.5%) patients who had met a student for a level 3 medication review and answering this question agreed or strongly agreed that they were far more likely to speak to a pharmacist about their medicines or their health following the medication review, while 11 (24.5%) were unsure.

It was calculated that for a full randomised controlled trial (RCT) to demonstrate an effect on HbA1c, 159 patients would be required in each arm of control and intervention to demonstrate 80% power with 214 patients per group to demonstrate a 90% power. Data utilised for sample size analysis are presented in table 3.

**Table 3:** Data used to calculate sample size

Output measure	Standard error of mean difference #	Standard deviation of mean difference	Clinically important difference	Unit of clinical measure	Number of patients required in each group
HbA1c	2.28	17.5	5.5	mmol/mol	159
Systolic blood pressure	2.27	12.47	3.3	mm Hg	224

Patients were included in the intention to treat analysis even if they did not complete questionnaires.

HbA1c, glycated haemoglobin.

### Health economics analysis

The mean per participant cost of the intervention was £164.41; table 4 provides a breakdown of the component parts. We used different versions of the EQ-5D (the five-level version^38^ at baseline and the three-level^39^ at follow-up) to assess whether completion rates differed according to the number of levels. The response rates were slightly higher for the EQ-5D-3 L and the mean change in EQ-5D score was slightly higher for the intervention arm (see tables 1 and 2). Complete cost and effect data were available for 72 participants (54.1% of the sample).

**Table 4:** Main points learned from stakeholder meetings

Location	Patients	Students
Preintervention	Care plan or protocol for use during consultation Current unmet information need Ensure paperwork is easily comprehensible Ensure students are confident Ensure preparative training Supervision of student essential Students must admit knowledge gaps	Transport should be provided Group feedback on their performance where possible
Postintervention	Real-life teaching required Willingness to participate in the future Recruitment—avoid Christmas and mail best method Confirmed GP practice best location Praise (not universal) for students’ consultation skills Request use of email within consent and utilise for appointments and reminders where agreed	Preparative training—some elements and timing criticised. Role play most effective Supervisor feedback useful Level 2 medication effective preparation for consultation Wanted consultations with more patients Difficulties completing study outside course Educational benefit obtained Preferred to Objective Structured Clinical Examinations (OSCEs) or role play
Location	Primary care pharmacists	Medical practice staff
Preintervention	Care plan or protocol for use during consultation Ensure preparative training Students access medical record prior to consultation	Care plan or protocol for use during consultation Ensure preparative training Students access medical record prior to consultation Ensure students do not contradict nurse advice
Postintervention	Role play good—in ‘protected’ environment Supervisor feedback useful Enjoyed participation Improved their own continuing professional development (CPD) Confirmed that GP practice best location Feedback after each consultation improved performance	Consultation must be recorded in patient's records Need protected time for students to feedback recommendations

GP, general practitioner.

### Stakeholder meetings

All stakeholder groups supported the testing of the idea. There was agreement that student-led consultations should take place at the medical practice of each patient rather than at the university, should not be time-limited and should be supervised. Postintervention stakeholder groups agreed that the experience had been generally positive, had provided both student and patient benefit and raised awareness of the role of pharmacists.

Table 4 provides a summary of the main points learned from the stakeholder meetings before and after intervention where opinions did not concur across groups.

Table 5 presents identified intervention costs. It can be seen that there are significant fixed costs associated with setting up the service: the mean cost per participant is relatively small.

**Table 5:** Resource utilisation costs

Component part	Resources costed (unit cost), participant costing	Total cost (£)	Mean cost as £ per participant
Development of training plan	1 h meeting for 4 people (3 pharmacists @ £50 per hour*; 1 RA @£25 per hour†)	175.00	2.61
Development of background material	Podcasts on diabetes and cardiovascular and communication skills. 1.5 h of pharmacist time @ £50 per hour*;	75.00	1.12
IT workshop	1 day preparation (RA @£25 per hour†); IT Dept. costs (room and trainer for 4*0.5 day sessions—£600 flat fee); supervision and assistance with training (2 days of pharmacist time @£50 per hour*)	887.50	13.25
Care planning workshop	Preparation (1 h RA @£25 per hour† and 1 h pharmacist @ £50 per hour); delivery (3 h RA time @£25 per hour† and 3 h pharmacist time @ £50 per hour)	300.00	4.48
Communication/consultation skills with motivational interviewing	Preparation (2 h pharmacist time @£50 per hour); delivery (3 h RA time @£25 per hour† and 3 h pharmacist time @ £50 per hour)	325.00	4.85
Role play workshop	Preparation (9.5 h RA time @£25 per hour† and 3 days of pharmacist time @£50 per hour) Per session: 2 h of consultation/MR practice (2 h RA time @£25 per hour† and 2 h pharmacist time @ £50 per hour) and 1 h of GP feedback (1 h RA time @£25 per hour† and 1 h GP time @ £118 per hour). 7 sessions (6 students per session).	3238.50	48.34
Level 2 review	Based in general practice, look at medical records and create care plans. Per session: 3 hours of pharmacist time (@£50 per hour*). Specialist nurse attended for the last hour (@£52 per hour). 13 sessions held (3 students per group)	2626.00	39.19
Level 3 review	1 h per patient at general practice (pharmacist @£50 per hour*). Specialist nurse attended for 15 min (@£52 per hour). Held with 54 patients	3402.00	50.78

*Taken from Curtis.^37^

†Estimate based on within study costs.

GP, general practitioner; IT, information technology; MR, medication review.

## Discussion

This pilot study has demonstrated the acceptability and practicality of pharmacy students providing full medication review to patients with type 2 diabetes under supervision. All stakeholder groups displayed support for the concept, with patients also displaying a willingness to participate in a subsequent RCT. Medical practices were purposively selected, and no problems were experienced with patient recruitment, though recruitment rates were relatively low (17%) and may display a better response rate without postal issues experienced at one medical practice. Nevertheless, future recruitment for a full RCT would be possible and may display a better response without postal issues experienced at one medical practice. Importantly, there was also a very low patient dropout rate (<10%). The logistics of patients having to attend an additional clinic within the intervention group resulted in the loss of patients due to illness and bereavement which were unavoidable losses to the study and two due to forgetting to attend. A longer period to allow for rebooking and reminders closer to the time of the appointment may have prevented these drop-outs. A longer time period to allow for rebooking and reminders closer to the appointment may have helped but would increase the costs. Potential patient benefit was also identified within some of the outcome measures. The results, therefore, provide evidence that within an RCT, sufficient patients could be expected to agree to attend for a medication-related consultation.

As this was a pilot study, and had small participant numbers we did not expect to demonstrate significant effects in our outcome measures. Students were volunteers and may not represent a full population of undergraduate pharmacy students. All participating medical practices were requested to start recruitment at the same time; however, results demonstrate patient recruitment proceeding over a period of greater than 4 months, resulting in logistical issues within the project. Ethical reasons required recruitment to be initiated via the medical practices, but results suggest that closer communication and support may be required between the researcher and medical practices to facilitate earlier recruitment. Written appointment information may aid patient attendance and prevent the small number of patients failing to attend due to forgetfulness. The large number of statistical tests carried out, increases the likelihood of false positives. Conversely as a pilot and feasibility study with limited sample size there is an increased likelihood of false negatives.

While student willingness to participate was high, pressures of concurrent timetabled course work and a lack of confidence in ability to perform the consultation resulted in significant drop out during the progress of the study. Integration of the service into the curriculum did not result in drop out from similar non-UK studies.^18^ ^19^ Participating students were volunteers and, therefore, self-selecting. Given that they were, on average, academically superior when compared to non-participating students, they would potentially have performed better than non-participants during a student-led consultation. Most studies do not mention this effect and any future study utilising volunteer students should recognise and allow for it.^18^ ^40–43^ The educational element of this study was not considered within this paper as this will form the focus of a future submission. The quality of medicines information given to patients affects individual's perception of whether it has met their needs and if they are satisfied with the information provided.^32^ It is reasonable to speculate that repetition of information over a period of years to patients with a long-term condition would have resulted in a greater understanding of and, therefore, satisfaction with information about their medicines. This may have reduced the ability to improve scores for many of the individual questions. While higher scores for SIMS is theoretically a predictor for better adherence (MARS)^32^ no improvement in adherence was observed. The relatively high proportion of patients who reported using medication compliance aids prior to the intervention might have reduced the potential for the intervention to further improve adherence. Adherence, beliefs about medication, satisfaction with diabetes treatment and quality of life all displayed a change in the direction which favours the intervention, all of which support a full study going ahead. No evidence is available to definitively explain the change in blood pressure seen in the control group and reasons for this would only be speculative, but it may simply have been a chance finding. It may be more appropriate to focus the intervention on those patients with the greatest HbA1c or the lowest reported satisfaction with information or adherence, however this may affect the recruitment rate and would require careful consideration prior to implementation in a full RCT if using the results from this study for it basis.

Results demonstrate that HbA1c would appear to be a sensible primary outcome measure for a future study. A sample size of 214 patients per group (428 in total) would be required to demonstrate a 90% power. This would equate to 107 students (two patient consultations per student), which is achievable within a full RCT undertaken over more than one school of pharmacy. However, recent National Institute for Health and Care Excellence (NICE) guidance recommends the utilisation of a cardiac risk measure (QRisk2)^44–46^ which represents a compilation of clinical data. Insufficient data is available to calculate a sample size for QRisk2, therefore, the pragmatic decision would be to utilise a primary outcome measure of HbA1c with QRisk2 possibly utilised as a secondary outcome measure.

Implementation of student activity described within this study is in line with current government agenda for pharmacy education.^47^ Evidence does not exist in the UK to support the training of undergraduate pharmacy students when undertaking activities with real patients, although examples exist outside the UK.^18^ ^19^ ^42^ This study does not provide definitive evidence for this, but does provide support for a future definitive RCT to test this hypothesis.

With a known patient recruitment rate and low dropout rate this demonstrates that if utilising the same protocol for a future RCT, not only can sufficient numbers of patients be expected to be recruited and retained. Medical practices were chosen because they all used the same software system for electronic medical records, as this would facilitate student training, however, utilising additional systems would increase the number of general practitioner practices available for recruitment. Changes may be warranted to patient selection criteria, as recruitment of patients who are not yet clinically stabilised may be more receptive to information regarding their medicines. NICE CG66^44^ recommends a blood pressure target level of <140/80 mm Hg for most people with type 2 diabetes, and <130/80 mm Hg for those at more particular risk. The latter group includes people with raised albumin excretion rate (microalbuminuria or worse), estimated glomerular filtration rate <60 mL/min/1.73 m^2^, those with retinopathy, and those with prior stroke or transient ischaemic attack. Data to enable such differentiation was not obtained within the pilot study and should be included in the design of a future definitive RCT. Provision of opportunities for students to undertake more than two consultations in future studies may demonstrate further student and patient benefit. Results provide evidence that within an RCT, sufficient patients could be expected to agree to attend for a medication-related consultation. Written appointment information may aid patient attendance and prevent the small number of patients failing to attend due to forgetfulness. Where acceptable to participants, email appointments and reminders for consultations may be effective. This pilot study confirms that the data required for evaluation of health economics is possible in a RCT. To address the rate of availability of cost and effect data, in any future study we would make every effort to ensure that baseline measures are completed prior to randomisation. The design of a definitive trial should ensure that medical practitioners receive feedback from the students to potentially increase the effectiveness of the intervention. The results from this study display good generalisation, as recruitment and the intervention followed existing scenarios where possible, however student academic ability may affect interpretation. Results support a future RCT as the intervention appeared to have the potential to improve blood glucose sugar control, quality of life and medicine information and these findings need more formal testing.
